# The effect of psychosocial interventions for sexual health in patients with pelvic cancer: a systematic review and meta-analysis

**DOI:** 10.2340/1651-226X.2024.24204

**Published:** 2024-04-29

**Authors:** Samuel Ask, Kristina Schildmeijer, Viktor Kaldo, Amanda Hellström

**Affiliations:** a Department of Health and Caring Sciences, Faculty of Health and Life Sciences, Linnaeus University, Kalmar, Sweden; b Department of Psychology, Faculty of Health and Life Sciences, Linnaeus University, Växjö, Sweden; c Centre for Psychiatry Research, Department of Clinical Neuroscience, Karolinska Institutet, & Stockholm Health Care Services, Region Stockholm, Sweden

**Keywords:** Cancer, meta-analysis, psychosocial interventions, sexual health, systematic review

## Abstract

**Aim:**

The aim of this systematic review and meta-analysis was to explore and evaluate the effect of psychosocial interventions in improving sexual health outcomes among post-treatment patients with pelvic cancer.

**Methods:**

Inclusion and exclusion criteria were pelvic cancer survivors; psychosocial interventions; studies with a control group and measures of sexual health. Five databases were searched for literature along with an inspection of the included studies’ reference lists to extend the search. Risk of bias was assessed with the RoB2 tool. Standardised mean difference (SMD) with a random effects model was used to determine the effect size of psychosocial interventions for sexual health in patients with pelvic cancers.

**Results:**

Thirteen studies were included, with a total number of 1,541 participants. There was a large heterogeneity regarding the type of psychosocial intervention used with the source found in a leave one out analysis. Six studies showed statistically significant improvements in sexual health, while three showed positive but non-significant effects. The summary effect size estimate was small SMD = 0.24 (95% confidence interval [CI]: 0.05 to 0.42, *p* = 0.01).

**Discussion:**

There is limited research on psychosocial interventions for sexual health in pelvic cancer patients. There are also limitations in the different pelvic cancer diagnoses examined. Commonly, the included articles examined physical function rather than the whole sexual health spectrum. The small effect sizes may in part be due to evaluation of psychosocial interventions by measuring physical dysfunction. Future research should broaden sexual health assessment tools and expand investigations to more cancer types.

## Background

Cancer is a global health concern, with the number of cases reported worldwide rising over the past few years and projected to continue to increase. By 2030, there are estimated to be 21.6 million new cases of cancer diagnosed each year [1]. Certain types of cancer, such as colorectal and prostate cancer, are among the top 10 causes of mortality in Sweden [2]. Cancer’s impact is profound, affecting physical, emotional, and psychological health. With improved treatments and screenings, global cancer survival rates are rising [3], shifting focus from survival to long-term quality of life, including sexual health [4–8].

Pelvic cancer includes cancers occurring in the organs located in the pelvic area, prostate, gynaecological organs (cervical, ovarian, uterine, vaginal, vulvar, and fallopian tube), colorectal (colon and/or rectum), and urinary bladder [9]. These cancers can significantly impact patients’ sexual health and well-being, leading to physical, emotional, and psychosocial issues [4–7]. Common sexual health disruptions, such as erectile dysfunction and dyspareunia, can stem from the diagnosis or treatments, particularly in pelvic cancer survivors [10].

There are several definitions of sexual health in the literature [11], however the most commonly used definition of sexual health is from World Health Organization (WHO) explaining several aspects of sexual health:

“A state of physical, emotional, mental and social well-being in relation to sexuality; it is not merely the absence of disease, dysfunction or infirmity.” [12]

Several factors affect sexual health in addition to cancer, such as depression and anxiety, which are psychological responses that are both symptoms and causes of decreased sexual health [4, 7]. Relationship with a partner is a key aspect for good sexual health [10], and intimate relationships can be a concern for patients with cancer [8]. The diagnosis may also affect partners in a negative way, with a loss or decrease of sexual activity and general intimacy. Single patients could experience a barrier for entering new intimate relationships due to the cancer diagnosis and treatment side effects [10]. The definition and right to sexual health is not exclusive to people in partner relationships [12]. The concept of sexual well-being, and therefore good sexual health, should be applicable to everyone, regardless of partner status [13].

Despite its prevalence, sexual health is often overlooked in cancer survivorship care, and patients may feel uncomfortable discussing their sexual concerns with healthcare providers, leaving these issues unattended [14]. The patients may also be in a treatment phase where they are not susceptible to interventions to improve sexual health, due to the potential focus on surviving [15]. There are implemented interventions aiming at physical sexual dysfunctions, while interventions aiming at psychosocial dimensions are less validated [16]. It is known that depression and anxiety affect sexual health, as does life circumstances such as being in a relationship or not.

Psychosocial interventions may be a vital part of the care for patients with pelvic cancer regarding sexual health problems. A psychosocial intervention is a non-pharmacological intervention involving interpersonal relationships between individuals or groups for example cognitive behavioural therapy, psychoeducation, psychotherapy, counselling, and supportive therapies [17]. These interventions aim to improve the patients’ mental and emotional health, thereby their overall quality of life [18, 19].

This review of the present research will investigate the effectiveness of psychosocial interventions for improving sexual health outcomes in patients with pelvic cancer. The information could be used to inform the potential development of more effective and targeted psychosocial interventions for this patient population, ultimately improving their overall quality of life.

## Aim

The aim of this systematic review and meta-analysis was to evaluate and explore the effect of psychosocial interventions in improving sexual health outcomes among post-treatment patients with pelvic cancer.

## Methods and materials

### Design

This systematic review and meta-analysis followed the Preferred Reporting Items for Systematic Reviews and Meta-analysis (PRISMA) statement [20]. The search terms and strategies followed the Population, Intervention, Control and Outcome (PICO) format [21], and were selected with guidelines from librarians. Using PICO, we identified Medical Subject Headings (MeSH) terms, major headings, and main subjects, with additional free text variations specific to each database. The population (P) includes all diagnoses categorised under pelvic cancer. The intervention (I) had to be psychosocial. Only Randomized controlled trial (RCT) studies with control group (C) were included. The outcomes (O) pertained to sexual health and function. Databases PubMed, CINAHL, PsycINFO, Cochrane library, and Assia were searched to identify relevant articles.

### Identification

The search was executed by authors SA and AH. The references of included studies were scanned for potential inclusion. No limitations or filters were set in any database. For detailed search history, see Supplementary Table 1.

### Screening

The selection process started with reading of the title and abstract of all identified studies after excluding duplicates (*n* = 597). Duplicate data were automatically identified using two independent reference management programmes and then removed manually. Exclusion and inclusion were done using a computer programme for organising and managing articles that allowed blinding between authors SA and AH in the screening phase. Discrepancies between the authors detected when the blinding was removed were solved via discussion. A third author, KS, was ready to be called if consensus could not be reached. There were no conflicts of consensus.

### Data collection

An Excel spreadsheet was used to gather extracted data from the included studies. Examples of extracted data included descriptions of study sample, intervention, and outcome measures (Supplementary Table 2). Regarding intervention details, extracted data included duration and number of sessions; if there were any adherence assistance and homework aspects. See Supplementary Table 3 for detailed information extracted about the interventions.

### Eligibility criteria

In line with PICO, our study includes pelvic cancer survivors undergoing psychosocial interventions. Eligible studies must have a control group and measure sexual health using specific instruments. We exclude studies not focussed on pelvic cancer or those that didn’t isolate diagnosis specific data. Interventions solely affecting physical activity or those without varied delivery methods were omitted. Studies were required to be written in English. See Table 1 for more details.

**Table 1:** Eligibility criteria according to PICO.

PICO	Inclusion Criteria	Exclusion Criteria
Population	Post-treatment (surgery and/or chemotherapy and/or radiation therapy) patients and/or “survivors” with: Prostate cancer, gynaecological cancers (cervical, ovarian, uterine, vaginal, vulvar, and fallopian tube), colorectal cancer (colon and/or rectal) and bladder cancer. If there is a mix of diagnosis, where some of the diagnosis is not in the pelvic cancer definition in the same study, and there is a fair number of above-mentioned patients compared to the other diagnosis these will be included.	Studies examining only patients with other cancer diagnoses than pelvic cancers or if it is impossible to differentiate results for pelvic cancer among mixed diagnosis described in the inclusion criteria.
Intervention	Primary psychological, educational, cognitive, social and/or therapeutic interventions. No exclusion will be made based on delivery alternatives. Delivery alternatives in this sense include, for example face-to-face or web-based, group or individual interventions.	Interventions where the secondary effect is of psychological, educational, cognitive, social and/or therapeutic nature will be excluded. For example, physical activity interventions that may affect the physical well-being and are inferred to affect psychosocial well-being.
Comparison	Treatment as usual and waitlist control groups will be included. If the control is a modified version of the intervention and the differences are clear this can also act as comparator.	When compared between to interventions of the same potential magnitude, for example when comparing the same intervention delivered by different methods (for example online or face to face) without a control group will be excluded as the finding mainly shows effectives of the delivery method and not about the intervention itself.
Outcomes	Sexual health domain aspects and/or sexual function with instruments intended for that purpose, that is, not just an open question in a form. If instruments have subscales for sexual health domains and/or sexual functions and can clearly be differentiated as separate outcomes these will also be included.	Studies measuring only described secondary measures, for example quality of life, without sexual health domains or sexual function will be excluded.

### Quality appraisal and risk of bias

We used the Critical Appraisal Skills Programme (CASP) checklist [22] o assess the quality of selected studies. There are no established guidelines on how to score the appraised articles. The initial quality screening revealed clear differences between the points scored and the general quality of the articles. Based on this, we present the quality of the articles as excellent, high, or moderate, represented by 1, 2, and 3, respectively, in Supplementary Table 2. To be included in the review, articles must achieve at least 8 of the 11 points in CASP. A risk of bias analysis was done using Cochrane guidelines on risk of bias (RoB2) [23]. Quality appraisal and risk of bias analysis was done independently by authors SA and AH. The figures for the quality appraisal and analysis of biascan be found in the supplementary material.

**Table 2:** Study characteristics of included studies.

1st Author Year,	Country	Design	N (Mean age) + Gender	Diagnosis	Psychosocial aspects of interventions	Sexual Health outcome measure	Post-treatment Results (p-value)
**RCT**							
**Chambers et al, 2013**	Australia	Two-arm longitudinal RCT Control: Usual care	Intervention: 372 (63.34) Male Control: 368 (63.43) Male	PC	Counselling.	Treatment side effects (EPIC)	No statistically significant Positive effect
**Chambers et al, 2015**	Australia	Three-arm longitudinal RCT Control: Usual Care	Intervention: 62 Nurse & 63 Peer (62.70) Male Control: 64 (62.70) Male	PC	Counselling and support.	Sexual function (IIEF) Sexual self-confidence (Psychological Impact of Erectile Dysfunction)	Positive effects No p-value for these analyses.
**Duhamel et al, 2016**	United States of America	Two-arm pilot-RCT Control: Usual care	Intervention: 33 (56.73) Women Control: 37 (54.27) Women	RC	Education.	Sexual Function (FSFI)	No statistically significant Positive effect (*p* = 0.213).
**Karlsen et al, 2021**	Denmark	Two-arm Pre-test-post-test RCT Control: Usual care	Intervention: 16 (62.5) Control: 19 (63.4)	PC	Counselling and Psychoeducation.	Sexual function (IIEF)	No statistically significant Positive effect (*p =* 0.71)
**Lepore et al, 2003**	United States of America	Three-arm Longitudinal RCT Control: Usual care	Intervention: 84 Education only (64.8) & 86 Education and discussion (64.8) Male Control: 80 (65.6) Male	PC	Education.	Sexual function (UCLA Prostate Cancer Index)	Statistically significant positive effects (*p* < 0.05)
**Li et al, 2016**	China	Two-arm Pre-test-post-test RCT Control: Usual care	Intervention: 119 (46.13) Female Control: 107 (46.08) Female	GYN	Emotional management, education, and support.	Sexual function (FSFI)	Statistically significant Positive effects (*p* = 0.000).
**Mohammadi et al, 2022**	Iran	Two-arm Pre-test-post-test RCT Control: Waitlist	Intervention: 55 (40.4) Female Control: 55 (40.5) Female	GYN	Psychosexual support and counselling.	Sexual function (FSFI)	Intervention and control groups did not differ. (*p* = 0.525)
**Penedo et al, 2007**	United States of America	Two-arm Pre-test-post-test RCT Control: Enhanced usual care (half-day psychoeducational seminar)	Intervention: 53 (65.5) Male Control: 40 (65.5) Male	PC	CBT group stress management.	Sexual function (EPIC)	Statistically significant Positive effects (*p <* 0.001)
**Robertson et al, 2016**	United Kingdom	Two-arm longitudinal Pilot-RCT Control: Usual care	Intervention: 21 (64.15) Male Control: 22 (63.27) Male	PC	Psychoeducation and coping strategies.	Sexual function (EPIC)	Statistically significant Positive effects (*p =* 0.04)
**Schofield et al, 2020**	Australia	Two-arm Longitudinal RCT Control: Usual care	Intervention: 156 (57) Female Control: 158 (56) Female	GYN	Psychosocial consultation and support.	Sexual interest, sexual worry and global sexual satisfaction and function (SVQ)	Intervention and control groups did not differ. (*p* = 0.05)
**Schover et al, 2012**	United States of America	Three-arm longitudinal RCT Control: Waitlist	Intervention: 40 F2F (64) & 41 Online (64) Male Control: 48 Male	PC	Sexual counselling.	Sexual function (IIEF)	F2F: Statistically significant Positive effects (*p ≤* 0.0001). Online: Statistically significant Positive effects (*p* = 0.0040).
**Skolarus et al, 2019**	United States of America	Two-arm Longitudinal RCT Control: Enhanced usual Care	Intervention: 278 (67.2) Male Control: 278 (66.2) Male	PC	CBT and coping framework.	Sexual Function (EPIC)	No statistically significant differences in outcome (*p* = 0.6)
**Wittman et al, 2022**	United States of America	Two-arm Longitudinal RCT Control: Usual care	Intervention: 62 (62) Male Control: 80 (61) Male	PC	Psychosexual support.	Sexual function (EPIC) Sexual interest (PROMIS)	No statistical differences for sexual interest or function (*p* = 0.8)

CBT: Cognitive Behavioural Therapy; CRC: Colorectal Cancer; EPIC: Expanded Prostate Cancer Index Composite; FSFI: Female Sexual Function Index; GYN: Gynaecological Cancer; IIEF: International Index of Erectile Function; PC: Prostate Cancer; RC: Rectal Cancer; SVQ: Sexual Function-Vaginal Changes Questionnaire.

### Data analysis and synthesis

The analysis was carried out using R (version 4.2.2) [24] and the metafor Package (version 3.8.1) [25]. We calculated the effect size using a standardized mean difference (SMD) measure with a random effects model because of the heterogeneity of measurements for sexual health. We used the first post-treatment measure, which is the first measure taken after the interventions ended. For one study [26], calculations of standard deviation (SD) were extracted by calculating standard error (SE) from the number of participant and 95% confidence intervals (CIs) using calculation SE = (upper limit – lower limit) / 3.92, and then using Cochrane calculation of SD = SE × sqrt(n) [21]. Cohens rule of thumb regarding effect size was used in this study where 0.1, 0.3 and 0.5 indicate small, medium and large effect sizes respectively [27].

The amount of heterogeneity was estimated using the restricted maximum-likelihood estimator Tau^2^ [28] with addition of a Q-test for heterogeneity [29]. The extent if variation across included studies, that is, percentage heterogeneity, are presented with *I*^2^ statistics where corresponding values are attributed to 0–40% (might not be important), 30–60% (may represent moderate heterogeneity), 50–90% (may represent substantial heterogeneity), and 75–100% (considerable heterogeneity) [21, 30].

Selection and small study bias were analysed using funnel plot with Eggers regression for a measure of asymmetry. A funnel plot displays the “true” effect size against several other factors such as sample size and standard error for example.

## Results

**Figure 1:**
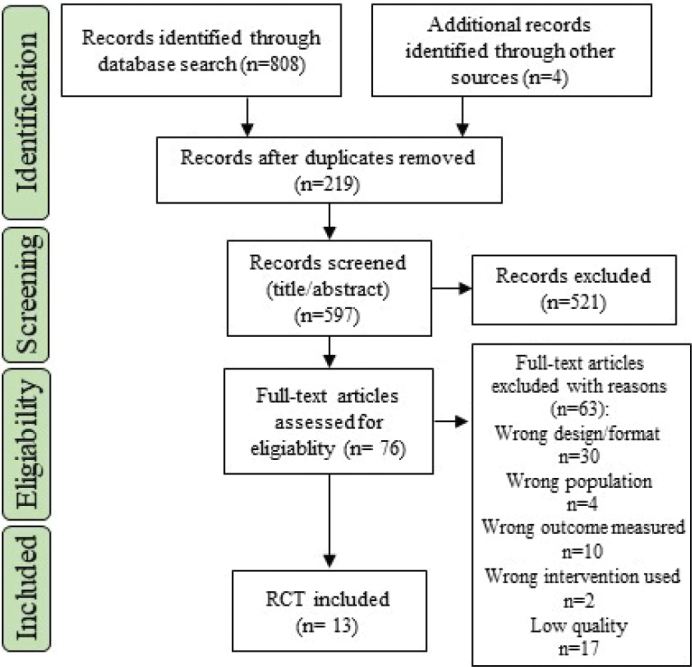
Flowchart of included studies.

A total of 812 articles, were identified. Four studies were identified via screening of the reference lists of included studies, of which two were included in the meta-analysis [31, 32] (Figure 1). There were 13 RCTs, [26, 31–42] including two 3-arm intervention studies identified with separate ID in the analysis [32, 34]. A total of 1,541 participants, were included with interventions in the meta-analysis.

The publication year ranged from 2003 to 2022 in the studies. The number of studies examining psychosocial interventions based on diagnosis were 9 prostate cancer studies (*n* = 1,178), 3 gynaecological cancer studies (*n* = 330), and 1 rectal cancer study (*n* = 33). All the studies examining gynaecological cancers focussed on cervical cancer. Some also included endometrial cancer [37, 40] and one also focussed on vaginal, vulvar, ovarian, and fallopian locations in addition [40]. No included studies examined the effects of psychosocial interventions on patients with bladder cancer.

The mean-age for the sample in the included studies was 60.21 years. There were observable differences with a lower mean-age in patients with gynaecological cancers (47.84 years ), compared to the mean age in patients with prostate cancer (64.09 years).

All instruments used for measuring sexual health domains in the included studies measured sexual functioning, with a focus on physical function. The most common measurement instrument for sexual health in the studies was the Expanded Prostate Cancer Index Composite (EPIC) which was used in 6 of the 13 included RCTs. See Table 2 in this document and Supplementary Table 3 in supplementary materials for detailed about outcome measures.

### Risk of bias in included studies

The overall risk of bias regarding included studies is of some concern with 10 of the included studies showing moderate risk of bias and 3 showing low risk of bias according to the risk of bias assessment with RoB2. More details on the risk of bias analysis are provided in the supplementary Figure 1.

### Type of psychosocial interventions

There was a variety and combination of different psychosocial components in interventions of nearly all included studies, necessitating the construction of categories. See Supplementary Table 3 for details about the different psychosocial components and treatment delivery methods in the interventions. The description of usual care was limited in all studies, making it difficult to generate inferences about potential comparative effects.

The mean number of sessions was 5.27 across all studies that disclosed the number of sessions. In studies where the number of sessions depended on recruitment time [34, 40], the lowest number was used. Only one study failed to specify the number of sessions of the intervention [36]. The mean duration for interventions were 16.46 weeks for the included studies.

Statistically significant improvements for sexual health domains, were presented in six of the included studies, five of which focussed on prostate cancer and one on gynaecological cancer [31, 32, 36, 38, 39, 42]. Non-statistically significant positive effects in sexual health were seen in three studies [33–35]. Four studies showed no differences between intervention and control [40] and three [26, 37, 41] of them had non-statistically significant results. Among the studies indicating no effect, all were published between 2019 and 2022; whereas there was only one study published after 2016 among those showing positive effects. There was no clear observable trend between the type of psychosocial intervention and a positive outcome, based on the categorisation in this study. There was no observable trend that face-to-face, web-based, or telephone-based interventions, were more effective than the other. Six of the interventions were couple based [26, 32, 34, 39, 41, 42] and three of these showed positive effects on sexual health outcomes with statistical significance. Of the seven [26, 34, 37, 39–42] studies with pre-registration, three [39, 40, 42] presented statistically significant results and one of these presented no positive effect [40]. Four studies measured the sexual health of the partner in addition to the patient [26, 32, 34, 42].

### Effectiveness of psychosocial interventions

**Figure 2:**
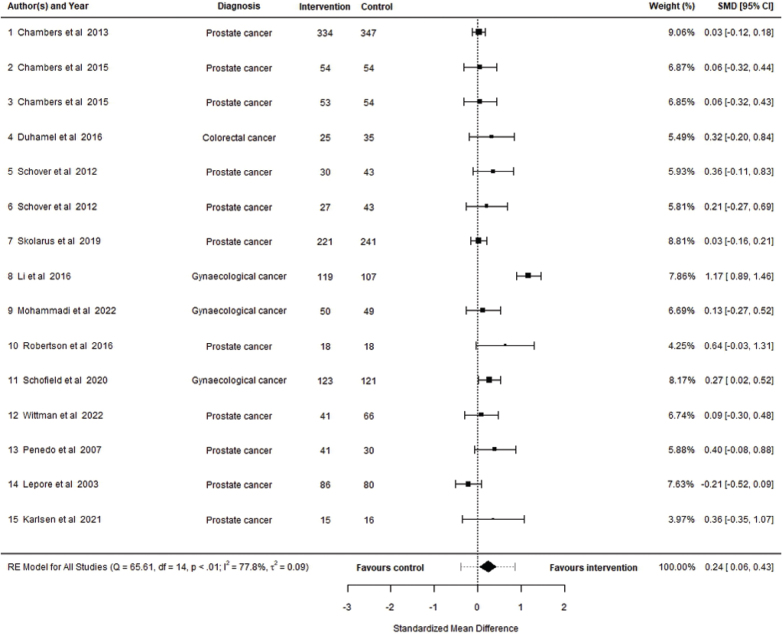
Forest plot showing the observed outcomes and the estimate of the random effects model.

A total of 15 interventions described in 13 studies were included in the meta-analysis. The observed SMD ranged from −0.2120 to 1.1729 with 93% of their estimates favouring the intervention. Based on the random effects model, the estimated SMD was 0.2414 (95% CI: 0.0569 to 0.4259, *p* = 0.01) with a statistically significant difference between outcome and zero (*p* = 0.0103), indicating a small post-intervention effect on psychosocial interventions for patients with pelvic cancer compared to control. A forest plot showing the observed outcomes and the estimate based on the random-effects model is shown in Figure 2.

There was significant heterogeneity in the true effects across the included studies according to the *Q*-test (*Q* = 65.6114, *p ≤* 0.0001). Approximately 78%, (*I*^2^ *=* 77.79%) of the variability of effect sizes can be attributed to the heterogeneity. There was a moderate amount of variance across included studies ($\overset{\wedge 2}{\tau }$ = 0.0919). A 95% prediction interval for the true outcomes is given by -0.3807 to 0.8635. Hence, although the average outcome is estimated to be positive, in some studies the true outcome may in fact be negative.

A moderator analysis for the potential influence of baseline differences indicated the presence of residual heterogeneity (*QE =* 66.1266, *p ≤* 0.0001, $\overset{\wedge 2}{\tau }$ = 0.0842, *I*^2^ *=* 78.05%) and a substantial proportion of variability across included studies is based on unaccounted sources. Further moderator analysis based on publication year (*QM =* 0.33, *p* = 0.56), mean-age of participants (*QM =* 0.25, *p* = 0.61), study quality (*QM =* 0.44, *p* = 0.50), type of control group (*QM =* 0.28, *p* = 0.96), intervention duration (*QM* = 0.84, *p* = 0.35), and if the intervention was couple based (*QM =* 0.51, *p* = 0.47), still indicated residual unaccounted heterogeneity albeit not statistically significant.

### Sensitivity analysis

**Figure 3:**
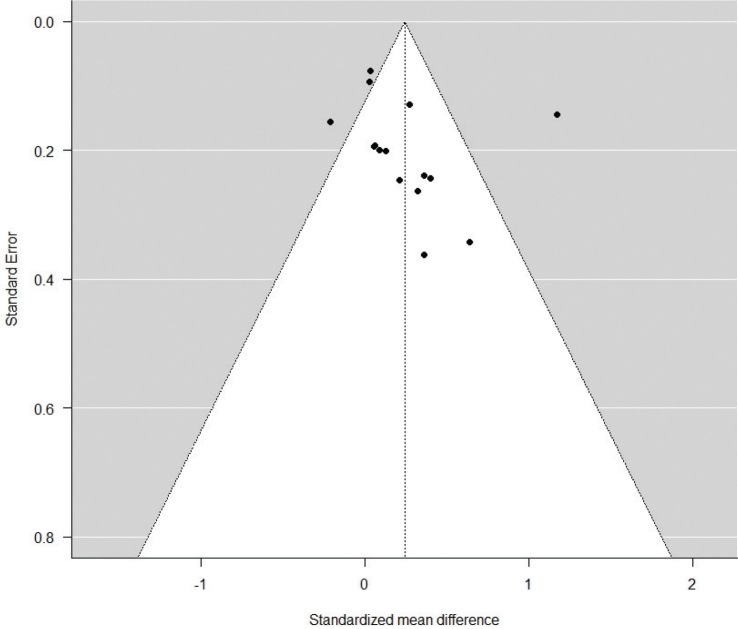
Funnel plot.

A funnel plot of the estimates is shown in Figure 3. The Eggers regression test did not display significant evidence of funnel plot asymmetry but indicated some degree of asymmetry based on the z-value (*z =* 0.8340, *p =* 0.4043). A rank correlation test was conducted which showed significant moderate positive correlation between effect sizes and study size (Kendall’s tau = 0.4286, *p* = 0.0275) indicating a moderate risk for small study bias.

**Figure 4:**
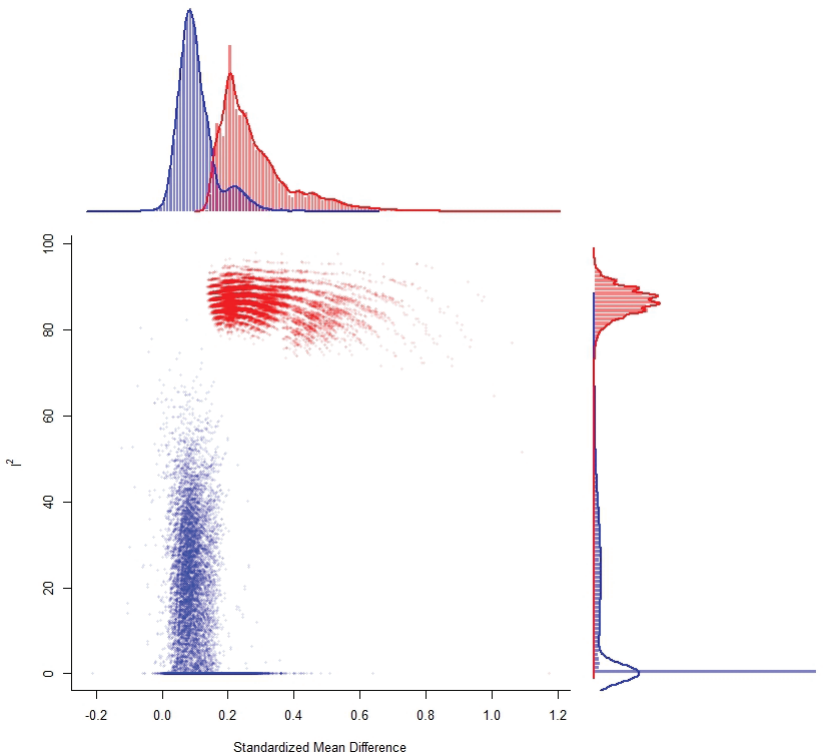
GOSH plot highlighting influence of study 8.

An examination of the forest and funnel plot revealed that one study showed signs of being a potential outlier in the context of this model. R student test indicated that study 8 [36], differed significantly from the other included studies. Using a GOSH plot the effect on heterogeneity for study 8, coloured red, is further verified and visualised with measuring all possible subsets of the data with fixed effects [43] (Figure 4).

A leave-one-out analysis was conducted, removing study 8 [36] from the analysis. This showed no significant amount of heterogeneity in the true outcomes (*Q* = 13.9079, *p* = 0.3804, Tau^2^ = 0.0028, *I*^2^ = 9.3154%), thereby indicating the source of the high heterogeneity score from the initial analysis and eliminating the need for further moderator analysis. The estimated SMD was 0.1028 (95% CI: 0.0120 to 0.1935, *p* = 0.0264) in the leave one out analysis.

## Discussion

The studies on psychosocial interventions for patients with pelvic cancer showed a variety in their components and delivery methods. The meta-analysis indicated a small but highly heterogeneous post-intervention effect, favouring the intervention, with no clear trend observed between the type of intervention and positive outcome.

Most studies included solely patients with prostate cancer, presenting difficulties in generalising about the pelvic cancer population in this context. The lack of research on gynaecological cancers was shown in this review. Sexual dysfunctions, both physical and psychosocial, are common in women with cancer, especially in the cases of cervical, breast and endometrial cancers [44]. One of the included studies in this review examined rectal or colorectal cancers with a small sample size. Colorectal cancers and its treatments have a major impact on sexual health [45, 46] thereby leaving the question of effectiveness of psychosocial interventions for these patients unanswered by this review. Although urinary bladder cancer has a significant impact on sexual health [7], this meta-analysis and review did not find any studies examining sexual health interventions in this group.

Psychosocial interventions included in this study are described in different ways regarding content and there were differences in what defines as a psychosocial intervention. Several interventions had various psychosocial components, making inferences of effects for specific components difficult. The heterogeneity of sexual health measures makes it difficult to draw conclusions about the effectiveness of the interventions. Almost all the included studies primarily measured sexual functioning (i.e. physical functioning) as the outcome for sexual health.

There was no observable trend that longer duration of interventions, intervention type and delivery modality were associated with more positive outcomes in this study. This may suggest that a longer intervention period does not necessarily lead to better sexual health outcomes. There may be positive effects from longer psychosocial interventions based on the depths of engagement, but too long durations may have a negative effect on adherence and fatigue. The same can be said about intensity of sessions [17].

There were a small, combined effect size indicating small differences between intervention and control groups. This suggests that the observed improvements may be due to temporal factors, such as time since treatment, rather than the intervention, since there were general improvements in both intervention and control group for studies showing statistically significant effects.

The studies reviewed varied widely in their methods of measuring outcomes related to sexual health, such as psychological distress and quality of life. This diversity prevented drawing conclusions about the impact of psychosocial interventions on sexual health. Additionally, few studies focussed on measuring the effect on sexual health for the partners, and no definitive conclusions could be drawn from them as these factors are known correlates [47]. The studies examining effects of interventions including partners were few and based on the aim and methods of this study, no fair inferences could be made. Relationship factors are linked to sexual health, but the current study could not derive conclusions about interventions targeting couples. Relationship satisfaction was measured in some studies, but the methods were inconsistent. These findings are in line with previous review literature [17].

The dysfunction is often caused by treatments leading to irreparable tissue damage, permanent nerve damage or endocrine and inflammatory changes [48]. A psychosocial intervention cannot repair this kind of damage. In line with the WHO definition of sexual health, it is important to consider more than just physical function when evaluating sexual health interventions, adding the psychological and social components to the physical aspect. This aspect together with measuring mostly physical dysfunction may be the cause for the small effect sizes presented in this meta-analysis. This is in line with previous meta-analysis, where effect sizes for psychological outcomes are reported as higher compared to quality of life (QoL) outcomes with psychosocial interventions, with albeit limited evidence [49].

Li et al. [36] was presented as an outlier in this study. Since all criteria and quality measures were met after a second round of controls, the study was included in the meta-analysis. However, the intervention delivery in Li et al. was sparsely described and the session time was not described. The intervention also included several evidence based multidisciplinary interventions in the patients’ home for a long duration that may yield favourable result albeit resource demanding. This study majorly contributed to the overall estimated effect size, leaving the combined effect size at a miniscule level after the leave-one-out analysis.

According to the funnel plot asymmetry verified by the moderate correlation of number of participants and effect size, there is a moderate risk of small study bias. Two studies [36, 39] reached a large effect size and four studies reached medium effect size [32, 35, 38, 42] based on Cohen’s rule of thumb, albeit containing a relatively small number of participants. There were negative values according to the CI in all studies presenting medium and large effects. There are no clear similarities between these studies on why the effect size is higher.

Over half (*n* = 7) of the RCT studies presented pre-registration protocols. All the RCT studies with pre-registration protocols were published from 2015 and later. This is in line with the steady increase in pre-registrations overall [50] since requirements were implemented in 2005 [51] and this is still not common to prospectively pre-register RCTs [52].

We decided not to use within group SMD, that is, compare the interventions groups pre- and post-test measures to generate effect size, since within group SMD disables the ability to measure the interventions effect due to the potential that a group without intervention (i.e. control) can experience the same benefits and not be presented [53]. This makes the between group SMD more appropriate when examining effects of interventions.

The included studies varied in significance regarding their individual results and all, but three studies showed some concern for bias, or a high risk of bias compared to low risk. The findings in this meta-analysis were statistically significant. However, the limitation in the included studies impacts the confidence in which inferences can be made about the effectiveness of psychosocial interventions for sexual health in patients with pelvic cancers.

The current study was pre-registered at PROSPERO. Deviations from the protocol entailed that a sub-group analysis based on cancer diagnosis, psychosocial intervention, and delivery methods were not possible due to the heterogeneity in the findings. Measuring long time effect size in the studies was not possible. No inferences could be made because of limitations and small amount of data leading to non-significant results for the heterogeneity statistics and the random effects model.

No confident recommendations for practice or policy can therefore be made based on the risk of bias, heterogeneity of measurements and interventions in the included studies. More RCTs are needed that use valid and comprehensive sexual health instruments that can better capture the effects of psychosocial interventions, rather than instruments only measuring physical function. The results of this study also show a lack of studies concerning patients with bladder, colorectal and gynaecological cancers. Research gaps have been identified, highlighting the necessity for dedicated studies on couple-based interventions to improve relationship outcomes. Additionally, there is a call for more in-depth research into the timing and duration of interventions to better understand their effects. The study of specific psychosocial components and the structure of interventions, particularly those that are couple-based and digitally delivered, is also needed. For interventions targeting couples, it is warranted to measure their effectiveness on both partners using validated methods.

As of this review it is difficult to discern inferences for specific components, but if the problem is based on several factors, one of which is physical, there may be a need for psychosocial interventions based on coping and self-efficacy in handling the new, permanent dysfunction, for example erectile dysfunction based on nerve damage.

## Supplementary Material

The effect of psychosocial interventions for sexual health in patients with pelvic cancer: a systematic review and meta-analysis

The effect of psychosocial interventions for sexual health in patients with pelvic cancer: a systematic review and meta-analysis

## Data Availability

The authors confirm that the data supporting the findings of this study are available within the article and its supplementary materials.
