# Supplementary material for: The effect of psychosocial interventions for sexual health in patients with pelvic cancer: a systematic review and meta-analysis
Source: Acta Oncol. 2024 Apr 29;68:24204. doi: 10.2340/1651-226X.2024.24204 (PMC11332557; doi:10.2340/1651-226X.2024.24204)

Supplementary material has been published as submitted. It has not been copyedited or typeset by Acta Oncologica.

Supplementary Figure 1 Risk of bias figures for included RCT studies

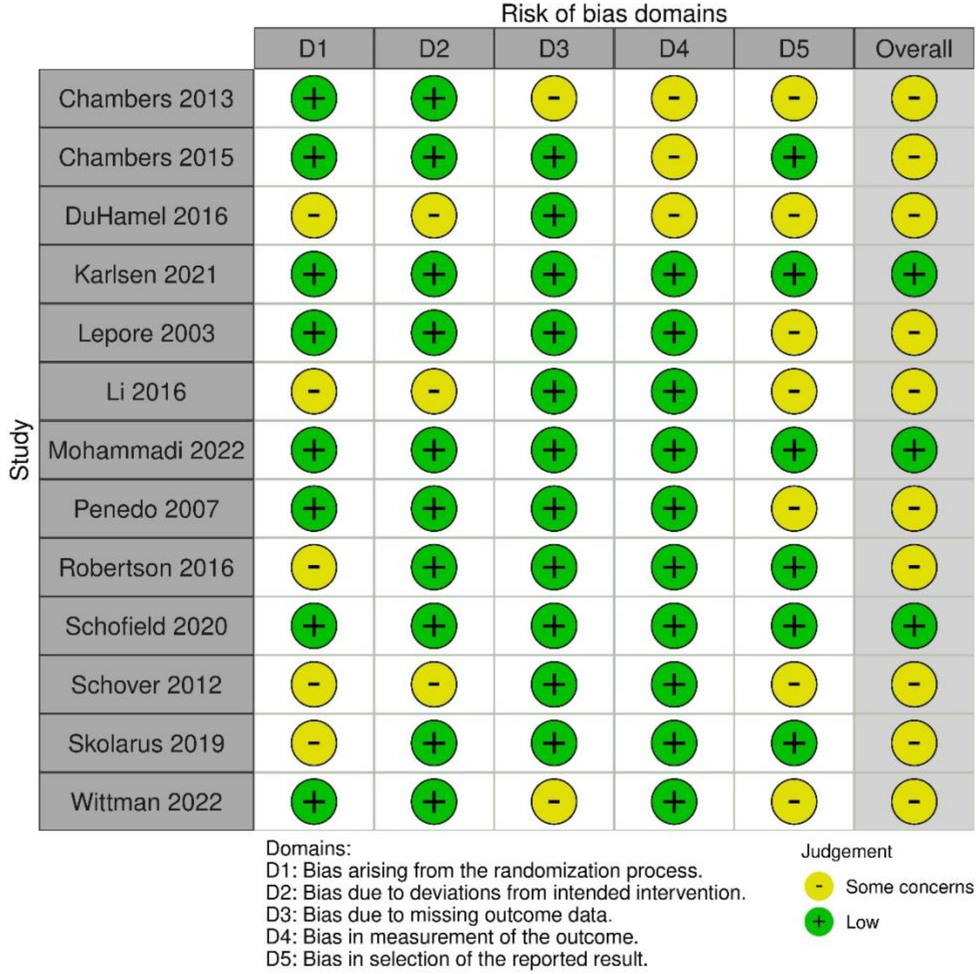

Supplement: The effect of psychosocial interventions for sexual health in patients with pelvic cancer: a systematic review and meta-analysis [file AO-63-24204-s1.pdf]
