# Supplementary material for: The effect of psychosocial interventions for sexual health in patients with pelvic cancer: a systematic review and meta-analysis
Source: Acta Oncol. 2024 Apr 29;68:24204. doi: 10.2340/1651-226X.2024.24204 (PMC11332557; doi:10.2340/1651-226X.2024.24204)
Supplement: The effect of psychosocial interventions for sexual health in patients with pelvic cancer: a systematic review and meta-analysis [file AO-63-24204-s2.pdf]

Supplementary material has been published as submitted. It has not been copyedited or typeset by Acta Oncologica.

Supplementary table 1 Database search history

Concept	Search terms	Results
**Database**: PubMed	**Date**: 2023-01-11	
1 Pelvic cancers	"Genital Neoplasms, Female"[MeSH Terms] OR "gynecologic neoplas*"[Title/Abstract] OR "gynecologic cancer*"[Title/Abstract] OR "gynecologic tumo*"[Title/Abstract] OR "gynecologic carcinom*" [Title/Abstract] OR "gynecological neoplas*"[Title/Abstract] OR "gynecological cancer*"[Title/Abstract] OR "gynecological tumo*"[Title/Abstract] OR "gynecological carcinom*" [Title/Abstract] OR "gynaecologic neoplas*"[Title/Abstract] OR "gynaecologic cancer*"[Title/Abstract] OR "gynaecologic tumo*"[Title/Abstract] OR "gynaecologic carcinom*" [Title/Abstract] OR "gynaecological neoplas*"[Title/Abstract] OR "gynaecological cancer*"[Title/Abstract] OR "gynaecological tumo*"[Title/Abstract] OR "gynaecological carcinom*" [Title/Abstract] OR "Urinary Bladder Neoplasms"[MeSH Terms] OR "bladder tumo*"[Title/Abstract] OR "bladder neoplas*"[Title/Abstract] OR "bladder cancer*"[Title/Abstract] OR "bladder carcinom*" [Title/Abstract] OR "Prostatic Neoplasms"[MeSH Terms] OR "Prostate neoplas*"[Title/Abstract] OR "Prostate cancer*"[Title/Abstract] OR "Prostate tumo*"[Title/Abstract] OR "Prostate carcinom*" [Title/Abstract] OR "Prostatic neoplas*"[Title/Abstract] OR "Prostatic cancer*"[Title/Abstract] OR "Prostatic tumo*"[Title/Abstract] OR "Prostatic carcinom*" [Title/Abstract] OR "Colorectal Neoplasms"[MeSH Terms] OR "colorectal neoplas*"[Title/Abstract] OR "colorectal tumo*"[Title/Abstract] OR "colorectal cancer*"[Title/Abstract] OR "colorectal carcinom*"[Title/Abstract] OR "Colonic Neoplasms"[MeSH Terms] OR "colonic cancer*"[Title/Abstract] OR "colonic tumo*"[Title/Abstract] OR "colonic neoplas*"[Title/Abstract] OR "colonic carcinom*"[Title/Abstract] OR "colon cancer*"[Title/Abstract] OR "colon tumo*"[Title/Abstract] OR "colon neoplas*"[Title/Abstract] OR "colon carcinom*"[Title/Abstract] OR "Rectal Neoplasms"[MeSH Terms] OR "rectal neoplas*"[Title/Abstract] OR "rectal cancer*"[Title/Abstract] OR "rectal tumo*"[Title/Abstract] OR "rectal carcinom*"[Title/Abstract] OR "rectum neoplas*"[Title/Abstract] OR "rectum cancer*"[Title/Abstract] OR "rectum tumo*"[Title/Abstract] OR "rectum carcinom*"[Title/Abstract]	793 618
2 Psychosocial intervetnyion	"Psychosocial Support Systems"[MeSH Terms] OR "social support*"[Title/Abstract] OR "psychosocial support*"[Title/Abstract] OR "psychological support*"[Title/Abstract] OR "Internet-Based Intervention"[MeSH Terms] OR "internet based intervention*"[Title/Abstract] OR "internet based intervention*"[Title/Abstract] OR "web based intervention*"[Title/Abstract] OR “Online-based intervention*”[Title/Abstract] OR "web based intervention*"[Title/Abstract] OR "online intervention*"[Title/Abstract] OR "internet intervention*"[Title/Abstract] OR "Behavior Therapy"[MeSH Terms] OR "behavior therap*"[Title/Abstract] OR “behaviour therap*”[Title/Abstract] OR "behavior treatment*"[Title/Abstract] OR “behaviour treatment*”[Title/Abstract] OR "conditioning therap*"[Title/Abstract] OR "behavior change technique*"[Title/Abstract] OR "behavior modification*"[Title/Abstract] OR “behaviour	178 270

	modification*”[Title/Abstract] OR “Cognitive therap*”[Title/Abstract] OR "Cognitive behavioral therapy"[MeSH Terms] OR "Cognitive behavioral therapy"[Title/Abstract] OR “Cognitive behavioural therap*”[Title/Abstract] OR "CBT"[Title/Abstract] OR "Psycho-Oncology"[MeSH Terms] OR "Psycho-Oncology"[Title/Abstract] OR "psychosocial oncology"[Title/Abstract] OR "psychooncology"[Title/Abstract] OR "psycho education*"[Title/Abstract] OR "psychoeducation*"[Title/Abstract] OR "Psychosexual"[Title/Abstract] OR "psycho sexual"[Title/Abstract] OR "sexual intervention*"[Title/Abstract]	
3 Sexual health	Sexual Health[MeSH Terms] OR “Sexual Health”[Title/Abstract] OR Psychosexual[Title/Abstract] OR “Psycho-sexual”[Title/Abstract] OR Sexual Dysfunctions, Psychological[MeSH Terms] OR “Sexual wellbeing”[Title/Abstract] OR “Sexual well-being”[Title/Abstract] OR “Sexual function”[Title/Abstract] OR “Sexual dysfunction”[Title/Abstract] OR “Sexual satisfaction”[Title/Abstract] OR Intimacy[Title/Abstract] OR Intimate[Title/Abstract] OR “Body image”[Title/Abstract] OR “Self-image”[Title/Abstract]	107 384
	1 AND 2 AND 3	359

**Database**: Cinahl	**Date**: 2023-01-11	
1 Pelvic cancers	(MH "Pelvic Neoplasms") OR TI ("pelvic neoplas*" OR "pelvic cancer*"OR "pelvic tumo*"OR) OR AB ("pelvic neoplas*" OR "pelvic cancer*"OR "pelvic tumo*"OR) OR TI ("pelvis neoplas*"OR "pelvis cancer*"OR "pelvis tumo*" OR "pelvis carcinom*") OR ("pelvis neoplas*"OR "pelvis cancer*"OR "pelvis tumo*" OR "pelvis carcinom*") OR (MH "Bladder Neoplasms") OR TI ("bladder tumo*"OR "bladder neoplas*" OR "bladder cancer*" OR "bladder carcinom*") OR AB ("bladder tumo*"OR "bladder neoplas*" OR "bladder cancer*" OR "bladder carcinom*") OR (MH "Genital Neoplasms, Female+") OR TI ("gynecologic neoplas*" OR "gynecologic cancer*" OR "gynecologic tumo*" OR "gynecologic carcinom*") OR AB ("gynecologic neoplas*" OR "gynecologic cancer*" OR "gynecologic tumo*" OR "gynecologic carcinom*") OR TI ("gynecological neoplas*" OR "gynecological cancer*" OR "gynecological tumo*" OR "gynecological carcinom*") OR AB ("gynecological neoplas*" OR "gynecological cancer*" OR "gynecological tumo*" OR "gynecological carcinom*") OR TI ("gynaecologic neoplas*" OR "gynaecologic cancer*" OR "gynaecologic tumo*" OR "gynaecologic carcinom*") OR AB ("gynaecologic neoplas*" OR "gynaecologic cancer*" OR "gynaecologic tumo*" OR "gynaecologic carcinom*") OR TI ("gynaecological neoplas*" OR "gynaecological cancer*" OR "gynaecological tumo*" OR "gynaecological carcinom*") OR AB ("gynaecological neoplas*" OR "gynaecological cancer*" OR "gynaecological tumo*" OR "gynaecological carcinom*") OR (MH "Prostatic Neoplasms+") OR TI ("Prostate neoplas*" OR "Prostate cancer*" OR "Prostate tumo*"OR "Prostate carcinom*") OR AB ("Prostate neoplas*" OR "Prostate cancer*" OR "Prostate tumo*"OR "Prostate carcinom*") OR TI ("Prostatic neoplas*" OR "Prostatic cancer*" OR "Prostatic tumo*" OR "Prostatic carcinom*") OR AB ("Prostatic neoplas*" OR "Prostatic cancer*" OR "Prostatic tumo*" OR "Prostatic carcinom*") OR (MH "Colorectal Neoplasms+") OR TI ("colorectal neoplas*" OR "colorectal tumo*" OR "colorectal cancer*" OR "colorectal carcinom*") OR AB ("colorectal neoplas*" OR "colorectal tumo*" OR "colorectal cancer*" OR "colorectal carcinom*") OR TI ("colonic cancer*" OR "colonic tumo*" OR "colonic neoplas*" OR "colonic carcinom*") OR AB ("colonic cancer*" OR "colonic tumo*" OR "colonic neoplas*" OR "colonic carcinom*") OR TI ("colon cancer*" OR "colon tumo*" OR "colon neoplas*" OR "colon carcinom*") OR AB ("colon cancer*" OR "colon tumo*" OR "colon neoplas*" OR "colon carcinom*") OR TI ("rectal neoplas*" OR "rectal cancer*" OR "rectal tumo*" OR "rectal carcinom*") OR AB ("rectal neoplas*" OR "rectal cancer*" OR "rectal tumo*" OR "rectal carcinom*") OR TI ("rectum neoplas*" OR "rectum cancer*" OR "rectum tumo*" OR "rectum carcinom*") OR AB ("rectum neoplas*" OR "rectum cancer*" OR "rectum tumo*" OR "rectum carcinom*")	8491

2 Psychosocial intervetnyion	(MH "Support, Psychosocial+") OR TI (“social support*” OR “psychosocial support*” OR “psychological support*”) OR AB (“social support*” OR “psychosocial support*” OR “psychological support*”) OR (MH "Psychosocial Intervention") OR TI “psychosocial intervention*” OR AB “psychosocial intervention*” OR (MH "Internet-Based Intervention") OR TI (“internet based intervention*” OR “web based intervention*” OR “online intervention*” OR “internet intervention*” OR “Online-based intervention*”) OR AB (“internet based intervention*” OR “web based intervention*” OR “online intervention*” OR “internet intervention*” OR “Online-based intervention*”) OR (MH "Behavior Therapy") OR TI (“behavior therap*” OR “behaviour therap*” OR “behavior treatment*” OR “behaviour treatment*” OR “conditioning therap*” OR “behavior change technique*” OR “behavior modification*” OR “behaviour change technique*” OR “behaviour modification*”) OR AB (“ behavior therap*” OR “behaviour therap*” OR “behavior treatment*” OR “behaviour treatment*” OR “conditioning therap*” OR “behavior change technique*” OR “behavior modification*” OR “behaviour change technique*” OR “behaviour modification*”) OR (MH "Cognitive Therapy+") OR TI (“Cognitive therap*” OR “Cognitive behavioural therap*” OR “Cognitive behavioral therap*” OR CBT) OR AB (“Cognitive therap*” OR “Cognitive behavioural therap*” OR “Cognitive behavioral therap*” OR CBT) OR (MH "Psycho-Oncology") OR TI (“psycho oncology” OR “psychosocial oncology” OR psychooncology) OR AB (“psycho oncology” OR “psychosocial oncology” OR psychooncology) OR (MH "Psychoeducation") OR Psycho-education* OR Psychoeducation* OR TI “sexual intervention*” OR AB “sexual intervention*”	171 638
3 Sexual health	(MH "Sexual Health") OR TI (“Sexual Health” OR “Sexual wellbeing” OR “Sexual well-being” OR “Sexual function” OR “Sexual dysfunction” OR “Sexual satisfaction” OR Intimacy OR Intimate OR “Body image” OR “Self-image”) OR AB (“Sexual Health” OR “Sexual wellbeing” OR “Sexual well-being” OR “Sexual function” OR “Sexual dysfunction” OR “Sexual satisfaction” OR Intimacy OR Intimate OR “Body image” OR “Self-image”) OR (MH "Sexual Dysfunction, Psychological") OR TI (Psychosexual OR “Psycho-sexual”) OR AB (Psychosexual OR “Psycho-sexual”)	47 641
	1 AND 2 AND 3	180
**Database:** PsycInfo	**Date**: 2023-01-11	
1 Pelvic cancers	"pelvic neoplas*" OR "pelvic cancer*" OR "pelvic tumo*" OR "pelvic carcinom*" OR "pelvis neoplas*" OR "pelvis cancer*" OR "pelvis tumo*" OR "pelvis carcinom*" OR "bladder tumo*" OR "bladder neoplas*" OR "bladder cancer*" OR "bladder carcinom*" OR "gynecologic neoplas*" OR "gynecologic cancer*" OR "gynecologic tumo*" OR "gynecologic carcinom*" OR "gynecological neoplas*" OR "gynecological cancer*" OR "gynecological tumo*" OR "gynecological carcinom*" OR "gynaecologic neoplas*" OR "gynaecologic cancer*" OR "gynaecologic tumo*" OR "gynaecologic carcinom*" OR "gynaecological neoplas*" OR "gynaecological cancer*" OR "gynaecological tumo*" OR "gynaecological carcinom*" OR "colorectal neoplas*" OR "colorectal tumo*" OR "colorectal cancer*" OR "colorectal carcinom*" OR "rectal neoplas*" OR "rectal cancer*" OR "rectal tumo*" OR "rectal carcinom*" OR "rectum neoplas*" OR "rectum cancer*" OR "rectum tumo*" OR "rectum carcinom*" OR "colon cancer*" OR "colon tumo*" OR "colon neoplas*" OR "colon carcinom*" OR "colonic cancer*" OR "colonic tumo*" OR "colonic neoplas*" OR "colonic carcinom*" OR "Prostate neoplas*" OR "Prostate cancer*" OR "Prostate tumo*" OR "Prostate carcinom*" OR "Prostatic neoplas*" OR "Prostatic cancer*" OR "Prostatic tumo*" OR "Prostatic carcinom*"	8491

2 Psychosocial intervetnyion	MAINSUBJECT.EXACT.EXPLODE("Psychosocial Rehabilitation") OR tiab("social support system*" OR "psychosocial support*" OR "psychological support system*" OR "psychosocial intervention*") OR MAINSUBJECT.EXACT.EXPLODE("Digital Interventions") OR tiab("internet based intervention*" OR "web based intervention*" OR "online intervention*" OR "internet intervention*" OR "Online-based intervention*") OR MAINSUBJECT.EXACT.EXPLODE("Cognitive Therapy") OR tiab("behavior therap*" OR "behaviour therap*" OR "behavior treatment*" OR "behaviour treatment*" OR "conditioning therap*" OR "behavior change technique*" OR "behavior modification*" OR "behaviour change technique*" OR "behaviour modification*") OR MAINSUBJECT.EXACT.EXPLODE("Cognitive Behavior Therapy") OR tiab("Cognitive behavioural therap*" OR "Cognitive behavioral therap*" OR CBT) OR MAINSUBJECT.EXACT.EXPLODE("Psychoeducation") OR tiab(Psycho-education* OR Psychoeducation* OR "sexual intervention*")	145 556
3 Sexual health	MAINSUBJECT.EXACT.EXPLODE("Sexual Health") OR tiab("Sexual Health" OR "Sexual wellbeing" OR "Sexual well-being" OR "Sexual function" OR "Sexual dysfunction" OR "Sexual satisfaction" OR Intimacy OR Intimate OR "Body image" OR Self-image) OR MAINSUBJECT.EXACT.EXPLODE("Psychosexual Behavior") OR tiab(Psychosexual OR "Psycho-sexual")	302 706
	1 AND 2 AND 3	118
**Database:** ASSIA	**Date**: 2023-01-11	
1 Pelvic cancers	(MAINSUBJECT.EXACT.EXPLODE("Colon cancer") OR MAINSUBJECT.EXACT.EXPLODE("Cervical cancer") OR MAINSUBJECT.EXACT.EXPLODE("Bladder cancer") OR MAINSUBJECT.EXACT.EXPLODE("Colorectal cancer") OR MAINSUBJECT.EXACT.EXPLODE("Gynaecological cancer") OR MAINSUBJECT.EXACT.EXPLODE("Pelvic cancer") OR MAINSUBJECT.EXACT.EXPLODE("Ovarian cancer") OR MAINSUBJECT.EXACT.EXPLODE("Prostatic cancer")) OR (title((pelvic OR pelvis OR bladder OR gynecologic OR gynaecologic OR gynecological OR gynaecological OR colorectal OR rectal OR rectum OR colon OR colonic OR prostate OR prostatic) PRE/1 (cancer* OR neoplas* OR tumo* OR carcinom*)) OR abstract((pelvic OR pelvis OR bladder OR gynecologic OR gynaecologic OR gynecological OR gynaecological OR colorectal OR rectal OR rectum OR colon OR colonic OR prostate OR prostatic) PRE/1 (cancer* OR neoplas* OR tumo* OR carcinom*)))	7113
2 Psychosocial intervetnyion	(MAINSUBJECT.EXACT.EXPLODE("Psychosocial support") OR MAINSUBJECT.EXACT.EXPLODE("Psychological intervention") OR MAINSUBJECT.EXACT.EXPLODE("Psychosocial therapy") OR MAINSUBJECT.EXACT.EXPLODE("Behaviour therapy") OR MAINSUBJECT.EXACT.EXPLODE("Cognitive behavioural psychotherapy") OR MAINSUBJECT.EXACT.EXPLODE("Psychoeducational treatment")) OR ((title("social support*" OR "psychosocial support*" OR "psychological support*") OR abstract("social support*" OR "psychosocial support*" OR "psychological support*")) OR (title("psycho oncology" OR "psychosocial oncology" OR psychooncology) OR abstract("psycho oncology" OR "psychosocial oncology" OR psychooncology)) OR ((title("psychosocial intervention*") OR abstract("psychosocial intervention*")) OR (title("sexual intervention*") OR abstract("sexual intervention*"))) OR (title("behavior therap*" OR "behaviour therap*" OR "behavior treatment*" OR "behaviour treatment*" OR "conditioning therap*" OR "behavior change technique*" OR "behavior modification*" OR "behaviour change technique*" OR "behaviour modification*") OR abstract("behavior therap*" OR "behaviour therap*" OR "behavior treatment*" OR "behaviour treatment*" OR "conditioning therap*" OR "behavior change technique*" OR "behavior modification*" OR "behaviour change technique*" OR "behaviour modification*")) OR (title("Cognitive therap*" OR "Cognitive behavioural therap*" OR "Cognitive behavioral therap*" OR CBT) OR abstract("Cognitive therap*" OR "Cognitive behavioural therap*" OR "Cognitive behavioral therap*" OR CBT)) OR title("internet based intervention*" OR "web based intervention*"OR "online intervention*" OR "internet intervention*" OR "Online-	34 527

	based intervention") OR abstract("internet based intervention*" OR "web based intervention*"OR "online intervention*" OR "internet intervention*" OR "Online-based intervention"))	
3 Sexual health	(MAINSUBJECT.EXACT.EXPLODE("Sexual health") OR MAINSUBJECT.EXACT.EXPLODE("Sexual dysfunction")) OR ((title("Sexual Health" OR "Sexual wellbeing" OR "Sexual well-being" OR "Sexual function" OR "Sexual dysfunction" OR "Sexual satisfaction" OR Intimacy OR Intimate OR "Body image" OR "Self-image") OR abstract("Sexual Health" OR "Sexual wellbeing" OR "Sexual well-being" OR "Sexual function" OR "Sexual dysfunction" OR "Sexual satisfaction" OR Intimacy OR Intimate OR "Body image" OR "Self-image")) OR (title(Psychosexual OR "Psycho-sexual") OR abstract(Psychosexual OR "Psycho-sexual")))	19 540
	1 AND 2 AND 3	37
**Database**: Cochrane	**Date**: 2023-01-11	
1 Pelvic cancers	MeSH descriptor: [Pelvic Neoplasms] explode all trees OR MeSH descriptor: [Urinary Bladder Neoplasms] this term only OR MeSH descriptor: [Colorectal Neoplasms] explode all trees OR MeSH descriptor: [Prostatic Neoplasms] explode all trees OR MeSH descriptor: [Genital Neoplasms, Female] explode all trees OR ("pelvic neoplasm" OR "pelvic cancer" OR "pelvic tumor" OR "pelvic carcinoma"):ti,ab,kw (Word variations have been searched) OR ("bladder neoplasm" OR "bladder cancer" OR "bladder tumor" OR "bladder carcinoma"):ti,ab,kw (Word variations have been searched) OR ("colorectal neoplasm" OR "colorectal cancer" OR "colorectal tumor" OR "colorectal carcinoma" OR "colon neoplasm" OR "colon cancer" OR "colon tumor" OR "colon carcinoma" OR "colonic neoplasm" OR "colonic cancer" OR "colonic tumor" OR "colonic carcinoma" OR "rectal neoplasm" OR "rectal cancer" OR "rectal tumor" OR "rectal carcinoma" OR "rectum neoplasm" OR "rectum cancer" OR "rectum tumor" OR "rectum carcinoma"):ti,ab,kw (Word variations have been searched) OR ("Prostate neoplasm" OR "Prostate cancer" OR "Prostate tumor" OR "Prostate carcinoma" OR "Prostatic neoplasm" OR "Prostatic cancer" OR "Prostatic tumor" OR "Prostatic carcinoma"):ti,ab,kw (Word variations have been searched) OR ("gynecologic neoplasm" OR "gynecologic cancer" OR "gynecologic tumor" OR "gynecologic carcinoma" OR "gynaecologic neoplasm" OR "gynaecologic cancer" OR "gynaecologic tumor" OR "gynaecologic carcinoma"):ti,ab,kw (Word variations have been searched)	50502
2 Psychosocial intervetnyion	MeSH descriptor: [Psychosocial Intervention] explode all trees OR MeSH descriptor: [Internet-Based Intervention] explode all trees OR MeSH descriptor: [Behavior Therapy] this term only OR MeSH descriptor: [Cognitive Behavioral Therapy] explode all trees OR MeSH descriptor: [Psychosocial Support Systems] explode all trees OR MeSH descriptor: [Psycho-Oncology] explode all trees OR ("social support" OR "psychosocial support" OR "psychological support" OR "psychosocial intervention"):ti,ab,kw (Word variations have been searched) OR ("psycho oncology" OR "psychosocial oncology" OR psychooncology):ti,ab,kw (Word variations have been searched) OR ("Psycho-education" OR Psychoeducation):ti,ab,kw (Word variations have been searched) OR ("internet based intervention" OR "web based intervention" OR "online intervention" OR "internet intervention" OR "Online-based intervention"):ti,ab,kw (Word variations have been searched) OR ("behavior therapy" OR "behaviour therapy" OR "behavior treatment" OR "behaviour treatment" OR "conditioning therapy" OR "behavior change technique" OR "behavior modification" OR "behaviour change technique" OR "behaviour modification"):ti,ab,kw (Word variations have been searched) OR ("Cognitive therapy" OR "Cognitive behavioural therapy" OR "Cognitive behavioral therapy" OR CBT):ti,ab,kw (Word variations have been searched) OR ("sexual intervention"):ti,ab,kw (Word variations have been searched)	52972
3 Sexual health	MeSH descriptor: [Sexual Health] explode all trees OR MeSH descriptor: [Sexual Dysfunctions, Psychological] explode all trees OR ("Sexual Health" OR "Sexual wellbeing" OR "Sexual well-being" OR "Sexual function" OR "Sexual dysfunction" OR "Sexual satisfaction" OR Intimacy OR Intimate OR "Body image" OR "Self-image"):ti,ab,kw (Word variations have been searched) OR (Psychosexual OR "Psycho-sexual"):ti,ab,kw (Word variations have been searched)	14676

	1 AND 2 AND 3	119

Supplementary Table 2 Study characteristics of included studies (with extended results description and secondary outcome measures)

1st Author Year, (Journal)	Treatment methods	Outcome measure for participants (Instrument)	Results description	Study quality
**Chambers et al, 2013**<br><br>**(Psycho-Oncology)**	Surgery, Radio therapy, Hormone therapy and watchful waiting.	Cancer specific psychological distress (RIES)<br><br>Anxiety (Subscale of Memorial Anxiety Scale for Prostate Cancer).<br><br>Decision related distress (Decisional Conflict Scale-Revised)<br><br>Cognitive judgement (Constructed Meaning Scale)<br><br>Global QoL (Satisfaction with Life Measure)<br><br>HrQoL (SF-36)<br><br>Treatment side effects (EPIC)	The results were analysed in 3 sub-groups that was determined pre-randomization: *Younger, Higher education* and *income.*<br><br>Statistically significant improvement of the intervention especially for younger higher education men included outcomes *cancer-specific distress* and *mental health*. For younger with lower education was associated with decreases in *cognitive judgemental adjustment* over time.<br><br>Sexual bother worsened over time for older control men and younger lower education men. Urinary bother improved over time for the older control group. Bowel bother improved for the younger higher education group.<br><br>The younger sub-groups had higher sexual bother and urinary bother scores than the older at baseline.<br><br>Age and baseline sexual function are key attributes in determining outcomes.	1

**Chambers et al, 2015**<br><br>**(Psycho-Oncology)**	Surgery	Sexual function (IIEF)<br><br>Sexuality needs (Supportive Care Needs Survey)<br><br>Sexual self-confidence (Psychological Impact of Erectile Dysfunction)<br><br>Self-esteem (Masculine Self Esteem Scale)<br><br>Marital satisfaction (RDAS)	Compared to control there was significant improvements in ED medical treatment adherence for both groups.<br><br>No significant effects for sexual function, sexuality needs, sexual self-confidence, masculine self-esteem, marital satisfaction, or intimacy was found.<br><br>Comparing intervention start between pre-surgery and post-surgery there is a significant effect for sexual function and sexual self-confidence.	2
**Duhamel et al, 2016**<br><br>**(Journal of Cancer Survivorship)**	Surgery, Chemotherapy, Radio therapy	Sexual Function (FSFI)<br><br>Severity of cancer distress (IES-R)<br><br>Psychological distress (BSI)<br><br>QoL (EORTC-QLQ-C30)	Follow-up 1 4 months after baseline / intervention start:<br>Significant treatments effects were found whit intervention group having lower IES and better QLQ-C30 (EF). Sexual function results were not statistically significant but showed improvements compared to control.<br><br>Follow-up 2 8 months after baseline / intervention start: Improvements in QLQ-C30 (EF) remained statistically significant.<br><br>Generally, the effect sizes of the intervention were most predominant between baseline and the first follow-up and decreasing between follow-up 1 and 2.	3
**Karlsen et al, 2021**	Surgery	Sexual function (IIEF)	No significant effect of the intervention was found on erectile function or any other secondary outcomes, except for significantly increased use of erectile dysfunction (ED) treatment by patients in the intervention group at 8 months.	1

**(Sexual Medicine)**		Urinary function (EPIC-26)<br><br>Dyadic adjustment (DAS)<br><br>QoL (SF-36)<br><br>Anxiety (SCL-92)<br><br>Depression (MDI)<br><br>Self-efficacy (GSE)		
**Lepore et al, 2003**<br><br>**(Health Psychology)**	Surgery, Radio therapy, Chemotherapy	Sexual function (UCLA Prostate Cancer Index)<br><br>QoL (SF-36)<br><br>Depression (CES-D)	Depressive symptoms were low at baseline and did not change significantly between groups. The intervention improved outcomes physical functioning and sexual bother. Urinary and sexual function were not significantly improved by the intervention compared to control. Education and discussion intervention showed the most effective of the two. The intervention was generally effective in improving QoL for prostate cancer patients.	2
**Li et al, 2016**<br><br>**(European Journal of Oncology Nursing)**	Radio therapy	QoL (FACT-Cx)<br><br>Sexual function (FSFI)<br><br>Family function (FACES-II)	Statistically significant increases were observed in QoL, sexual function, adaptability and cohesion increased post-intervention for the intervention group.	2
**Mohammadi et al, 2022**<br><br>**(Supportive Care in Cancer)**	Surgery, Chemotherapy	Sexual function (FSFI)<br><br>Sexual QoL (SQOL-F)	There were positive changes in sexual function and sexual QoL in both the intervention and control group. There were more positive improvements in the intervention group regarding all areas in FSFI and SQOL-F but only the domains of *sexual desire, lubrication, orgasm, pain, overall sexual function* and *sexual relationship satisfaction* subscales reached statistical significance.	1

**Penedo et al, 2007**<br><br>**(International Journal of Behavioral Medicine)**	Surgery, Radio therapy	Sexual function (EPIC)<br><br>QoL (FACT-G)	Statistically significant improvements regarding QoL domains of physical- and emotional well-being and sexual function was observed for the intervention group compared with control group. In contrast the total well-being and sexual function score did not reach statistical significance for the control group.	3
**Robertson et al, 2016**<br><br>**(The Journal of Sexual Medicine)**	Surgery	Sexual function (EPIC)<br><br>Anxiety and Depression (HADS)<br><br>Family functioning (SCORE-15)	The intervention group had statistically significant improvements regarding sexual function between baseline and 4 months post-intervention. The differences in the control group for the same period did not reach statistical significance. Sexual function levels seemed to return to nearer baseline values 6 months post-intervention.<br><br>There was no observed statistically significant difference in anxiety, depression, emotional functioning, and relational functioning between the two groups. Although the mean values for the intervention group showed more improvements compared to control.	3
**Schofield et al, 2020**<br><br>**(Gynecologic Oncology)**	Surgery Chemotherapy, Hormone tharapy	Sexual interest, sexual worry and global sexual satisfaction and function (SVQ)<br><br>Supportive/sexuality care needs (SCNS-SF34-R)<br><br>Symtom distress (MSAS-SF-R)<br><br>Cancer specific QoL (FACT-G)<br><br>Anxiety and Depression (HADS)	Anxiety and depression scores did statistically significantly improve post intervention but with very low effect sized (*d* = .20 & .10).<br><br>Cancer specific QoL, sexual satisfaction and symptom distress did not differ significantly between intervention and control groups.<br><br>Sexuality need decreased in the intervention group and increased in the control group.	3

**Schover et al, 2012**<br><br>**(Cancer)**	Surgery, Radio therapy	Sexual function (IIEF)<br><br>Relationship satisfaction (A-DAS)<br><br>Current distress (BSI)	Both FF and WEB interventions had significant positive effects on participants sexual function and satisfaction compared to control. This remained up to 1 year post-intervention.<br><br>Marital happiness and overall distress did not significantly change over time (albeit there was no initial distress at baseline).	2
**Skolarus et al, 2019**<br><br>**(Journal of Clinical Oncology)**	Surgery, Radio therapy	Sexual Function (EPIC)<br><br>Percived efficacy (PEPPI)<br><br>Coping (Brief cope instrument)<br><br>QoL (RAND)	When choosing focus area most participants choose sexual and urinary health. Few chose bowel and general health.<br><br>No statistically significant improvements were observed regarding sexual function or confidence in symptom management between intervention and control group. Albeit no statistical significance there was an improvement in all 5 EPIC domains 5 months post-intervention compared to control.	2
**Wittman et al, 2022**<br><br>**(Cancer)**	Surgery Chemotherapy, Hormone tharapy	Sexual function (EPIC)<br><br>Sexual interest (PROMIS)<br><br>Satisfaction with life (GSSL)	Satisfaction with sex life did not significantly differ between intervention and control group 6 months post-intervention.<br><br>No statistical significance was observed regarding sexual function but the mean scores of EPIC improved in both groups.<br><br>A majority in the intervention group reported an increase in non-penetrative sexual activity at 68% compared to 53% in the control group.<br><br>Partners in the intervention group reported that sexual activity increased compared to partners in the control group.	1

BSI = Brief Symptom Inventory, CES-D = Center for Epidemiologic Studies Depression Scale, EORTC-QLQ-C30 = The European Organisation for Research and Treatment of Cancer Quality of Life Questionnaire, EPIC = Expanded Prostate Cancer Index Composite, FACT-G/Cx = Functional Assessment of Cancer Therapy – General/Cervix, FACES-II = Family Cohesion Scale, FSFI = Female Sexual Function Index, FSDS = Female Sexual Distress Scale, GSE = General Self-efficacy Scale, GSSL = Global Survey

on Sustainable Lifestyles, HADS = Hospital Anxiety and Depression Scale, IIEF = International Index of Erectile Function, MDI = Major Depression Inventory, MSAS-SF-R = Memorial Symptom Assessment Scale Short Form with Modified Response Format, PCa-QoL = The Prostate Cancer-Related Quality of Life Scale, PROMIS = Patient-Reported Outcome Measurement Information System, RDAS = Revised Dyadic Adjustment Scale, RIES = Revised Impact of Event Scale, SCORE-15 = Systematic Clinical Outcome and Routine Evaluation, SCL-92 = Symptom Check List 92, SCNS-SF34-R = Supportive Care Needs – Short Form 34, SF-36 = Short Form 36, SQOL-F = Sexual Quality of Life Female, SVQ = Sexual Function-Vaginal Changes Questionnaire.

Supplementary Table 3 Intervention Content for all included studies.

Study	Description	Post-treatment recruitment period	Guidance	Homework	Duration and sessions
**Chambers et al, 2013**	Phone delivered 5 individual sessions for 5 months.<br><br>Telephone counselling with a nurse counsellor aiming a low intensity psychological intervention.<br><br>Content of the counselling included: Decision support, challenging unhelpful cognition, psychoeducation, stress-reduction techniques and coaching about problem solving skills.<br><br>2 sessions pre-treatment with focus on decision support and stress management in preparation for the treatment.<br><br>2 sessions 3- and 7-weeks post-treatment aimed at adjustment to cancer, problem solving and coping with treatment side effects.	Between the day of diagnosis and before treatment start.	One booster calls 5 months post treatment addressing treatments side effects and further preparation for the future.	None described	5 sessions (22 weeks)
**Chambers et al, 2015**	Phone delivered couple-based nurse counselling or phone delivered peer support.<br><br>Pre-surgery recruitment consisted of 2 sessions pre-surgery and 4 sessions beginning 2 weeks post-surgery. 2 additional 16- and 22-weeks post-surgery.<br><br>Post-surgery recruitment consisted of 1 session 2 weeks after recruitment. 3 sessions 10 weeks post-recruitment. 2 additional sessions 16- and 22-weeks post-recruitment.<br><br>Nurse counselling intervention: Model after cognitive behavioural sex and couple's therapy deliviered by 2 experienced prostate cancer nurse counsellors.	Recruitment: Pre-surgery (74%)<br><br>Post - urgery/treatment (26%)<br><br>Those that received treatment: mean 142.90 (SD 106.8) days since surgery.<br><br>Those yet to receive treatment: were scheduled to have surgery in a	None described	Nurse counselling intervention: Behavioural homework: Increasing expression of affection and non-demanding sexual touch.<br><br>Peer support intervention: None	6 session (post-surgery recruitment)<br><br>8 sessions (pre-surgery recruitment)<br><br>(22 weeks)

	Peer support intervention: Led by peer support volunteers (n=15) who was prostate cancer survivors and had undergone a prostatectomy with at least 12 months since their last treatment.<br><br>Both interventions-based content on skill training in couples' communication, conjoint coping with content and material relevant to the early treatment stage. Audio-visual DVD enhanced the psychoeducation and sexuality education. The nurse led intervention was a professionally defined support role whereas the peer led intervention was more based on mutual support, education and sharing of experiences.<br><br>Content includes Education about prostate cancer, menopaus and sexuality. Challenging beliefs about prostate cancer, ageing and sexuality. Help in coosing medical treatments for ED and how to use these in sexual relationships.	span of mean 33.50 days (SD 32.00).			
**Duhamel et al, 2016**	Mixed phone or in-person individual intervention.<br><br>Therapist led Cancer Survivorship Intervention-Sexual Health (CSI-SH)<br><br>4 sessions in the intervention.<br>1: Overview of sexual health<br>2: Discussion of strategies to improv sexual function and well-being.<br>3: Education of effective communication to use with partner<br>4: Providing educational booklets referrals.	Mean 5.18 (SD 4.28) years since treatment.	Between each session there was a booster telephone call aimed at working through potential issues to promote adherence.	Between each session there was homework aimed at working through potential issues to promote adherence.	4 sessions á 1 hour (4 weeks)
**Karlsen et al, 2021**	Couple counseling: Up to six sessions<br><br>Sexual counselor lead intervention with psychoeducation, communication skills, and sensuality training	2-3 months after surgery.	None described	Video home-training program: A supplementary tool for the patients to practice PFMT at home with guidance from a video.	6 sessions á 1 hour (6 months)

	Pelvic floor muscle training (PFMT): Up to three sessions with a certified physiotherapist who instructs the patients on how to perform exercises to strengthen the pelvic floor muscles and reduce urinary incontinence and erectile dysfunction.<br><br>The intervention aims to improve the sexual and urinary outcomes and relationship function of men who undergo surgery for prostate cancer and their female partners.				
**Lepore et al, 2003**	The study included 2 face-to-face group interventions:<br>1: Education only<br>2: Education and discussion<br><br>Education only consisted of lectures with room for questions for the lecturer (10 minutes) but not between participants.<br><br>Education and discussion intervention had the same concept regarding education but allocated more time (45 minutes) for group discussions afterwards. Discussion was led by a male clinical psychologist.<br><br>The education covered several topics with a multi-disciplinary approach over sessions:<br>1: An overview of prostate cancer biology an epidemiology (Oncologist)<br>2: Control of physical side effects (Urologist)<br>3: Nutrition and cancer (Dietitian)<br>4: Stress and coping (Oncology nurse)<br>5: Relationship and sexuality (Clinical psychologist)<br>6: Follow up and future health concerns (Urologist)	Education only 51.1 (SD 23.0) days since treatment.<br><br>Education and discussion mean 47.9 (SD 21.9) days since treatment.	None described	Take home printed summary of the lectures.	1 session / week á 1 hours (6 weeks)
**Li et al, 2016**	Nurse led health promotion program (NLHP-HB) include 5 domains.<br>1: Establishing a multidisciplinary team. Specialist nurses, gynaecological doctors, physiotherapist, psychological consultant, dietitians, and physical therapist. The nurse acted as the leader and	After a first operation within 7 days post-surgery in inclusion criteria.	Home follow-up monitoring consisted of an online communication platform used to ask questions and get answers.	None described	Sessions are not described.<br>(26 weeks)

	communication where the rest of the team provided consultation.<br>2: Physiological rehabilitation: Pelvic floor training.<br>3: Emotional release management. Yoga exercises<br>4: Informal social support systems: Consist of surrounding family and friends Education about the disease, alleviation of worry and fear and encouragement is to be enhanced by the social support system.<br>5: Home follow-up monitoring.	No description of time since treatment.	Telephone follow-up was carried out every 2 weeks. With permission a home visit was available every 2 och 3 months.		
**Mohammadi et al, 2022**	Face-to-face group intervention.<br><br>Psychosexual counselling based with a cognitive behavioural approach. Built on the "EXPLISSIT" framework.<br><br>Each counselling sessions comprised of a discussion topics.<br>1: Participants talked about their sexual issues related to the disease. Information about the significance and necessity of addressing couples' sexual issues with focus on the impact of gynaecological cancer (GC). Providing suggestions for improvement of participants sexual relations.<br>2: Recapping the first session with feedback from the participant. Participant discuss changes experienced in the sexual function after GC diagnosis. Providing suggestions to improve sexual function with sex therapy techniques.<br>3: Recapping the second session with feedback from the participant.<br>Participants discuss changes in SQoL after GC diagnosis. Providing suggestions to improve SQoL with psychosexual techniques (improving *body image, strengthening sexual self-confidence* and *self-efficacy)*.<br>4: Recapping the third session with feedback from the participant. Participants discuss changes in QoL after	No description of time since treatment.	None described	None described	1 session / week á 60-90 minutes (4 weeks)

	GC diagnosis. Providing suggestions to improve SQoL with sex therapy (relaxation, focusing and systematic desensitization techniques).				
**Penedo et al, 2007**	Face-to-face group intervention.<br><br>Interventions was led by a PhD level licenced clinical psychologist. It contained around a 90 minutes didactic, educational portion and 30 minutes of relaxation training. The didactic portion was based on topics from the previous week's session.<br><br>Topics of the intervention included cognitive behavioural approaches to stress management (*identification of distorted thoughts, rational thought replacement, effective coping, anger management, assertiveness training and development of social support*), prostate cancer specific information and relaxation training (*deep breathing, progressive muscle relaxation and imigary*).	Mean 11 (SD 4.3) months since treatment.	None described	Weekly homework was set between each session. The aim of the homework was to reinforced the didactic portion of the intervention.	1 session / week á 2 hours (10 weeks)
**Robertson et al, 2016**	Face-to-face couple-based intervention.<br><br>The intervention was led by practitioners accredited with counselling or psychotherapy qualifications.<br><br>The intervention comprised of 6 sessions.<br>1: Getting to know the couple. Couples' own definition of current problems, cancer diagnosis. Information about partners role and support from wider family networks.<br>2: Couples communication style and relationship. Explore how love, support, understanding, companionship, and affection is conveyed.<br>3: Integrational patterns of illness, coping and affection. Discussions about family resilience, dyadic adjustment in illness, role of partner and family related to illness, how people in the family express intimacy.<br>4: Couple intimacy before and after cancer via psychoeducation promoting closeness and express	Mean 10.67 months since surgery	The significance of adherence to the intervention was stressed during preparatory training sessions. Midway through the intervention there was an Q and A highlighting the importance of adherence.	None described	6 sessions á 50 minutes (17 weeks)

	intimacy after treatment. Information about the place for medical treatments and sex therapy techniques.<br>5: Further exploration of areas that have raised during previous sessions.<br>6: Summarizing couples' accomplishments, future planning and relapse prevention.				
**Schofield et al, 2020**	Trained nurse-led face-to-face or telephone consultation individual.<br><br>Delivered pre-treatments, mid treatments (not for brachytherapy-alone patients), and 2 post-treatments.<br><br>Sessions:<br>1: Tour of the radiation facility followed by consultation regarding their top 3 concerns.<br>2: Discussion about radiation side effects and the use of vaginal dilators and pelvic floor exercises.<br>3: Patients concern for treatment completion discussed along with psycho-sexual recovery. Completion of a survivorship care plan.<br>4: Exploring potential concerns. Barriers to self-care addressed. Information about the importance of vaginal dilations reinforced.	Six months after radio therapy treatment.	Peer (trained other patients at least 2 years post-treatment) support was scheduled 1-2 weeks after each nurse-led session. Peers provided psychosocial support and encouragement to adherence.	None described	3-4 sessions á 30-60 minutes. (17 weeks)
**Schover et al, 2012**	Counselling About Regaining Erections and Sexual Satisfaction (CAREss).<br><br>2 individual versions, one face-to-face (FF) and one internet-based (WEB). The FF version received printed material from the WEB version except for videos and animations.	FF: Mean 17 (SD 14) months since treatment-<br><br>WEB: Mean 25 (SD 24) months since treatment-	WEB: E-mail and later telephone reminders was used. Participants had opportunities to e-mail therapist all the time.<br>Booster telephone calls was scheduled for both groups at 1 and 3 months discuss progress and treatment goals after completion.	Cognitive behavioural homework. Submission via a standardized report form online for the WEB and on paper for FF.<br><br>WEB: Therapist e-mailed feedback to participants.<br><br>FF: Therapist discussed homework in session.	3 sessions á 50-90 minutes. (12 weeks)
**Skolarus et al, 2019**	Couple-based intervention via automated telephone and tailored newsletter.	Mean 4.1 years (range, 1.1 to 8.0 years)	None described	Monthly newsletter.	4 sessions (17 weeks)

	Building Your New Intervention. An automated telephone intervention along with a tailored newsletter.<br><br>Participants was contacted each month for 4 months by the automated telephone system where assessment of symptoms using EPIC was done. Participants could choose and change focus area of the intervention based on the urinary, sexual, bowel or general health for self-management support. After this the participants received a tailored newsletter for the chosen area with information about the chosen symptom of self-management strategies. These strategies was based on cognitive behavioural therapy and coping framework. The tools included in the newsletter were *Understand symptoms, learning about the symptoms, how to manage the symptoms, how to get help for the symptoms* and *how to talk to the physician about the symptoms.*				
**Wittman et al, 2022**	Online couple-based intervention.<br><br>TrueNTH is an intervention which aims at sexual recovery after prostate cancer. Is is based on expert feedback, patient and partner focus groups, and usability testing. The interventions were tailored to the participant depending on treatment (surgery/radiation) and partner gender (female/male).<br><br>The intervention consisted of 6 modules.<br><br>Each model included:<br>- An introductory video of a patient, partner, and sex therapist.<br>- Educational content relevant to the stage of recovery.<br>- Suggested activities for couples to maintain emotional and sexual connections.<br>1: Preparation for sexual and emotional side effects of treatments.<br>2: Overview of sexual aids.	Baseline surveys were completed by patients 2 weeks before treatment, and follow-up assessments were conducted at 3 and 6 months after treatment	Between modules e-mails with strategies for concerns that arose from the session were sent out.	None described	The 6 models were accessible during a 30 week period.

	3: Preparation for sexual encounters.<br>4: Strategies for expanding a sexual repertoire.<br>5: Incorporation of the new sexual model into the daily life.<br>6: Guidance in communication with health care professionals about sexual concerns.	
